# Study on the Synthesis, Biological Activity and Spectroscopy of Naphthalimide-Diamine Conjugates

**DOI:** 10.3390/molecules19067646

**Published:** 2014-06-10

**Authors:** Zhi-Yong Tian, Jing-Hua Li, Qian Li, Feng-Lei Zang, Zhong-Hua Zhao, Chao-Jie Wang

**Affiliations:** 1 Institute of Chemical Biology, Henan University, Kaifeng 475004, China; E-Mails: Zangfenglei@sohu.com (F.-L.Z.); Zhaozhonghua@sohu.com (Z.-H.Z.); 2 Key Laboratory of Natural Medicine and Immuno-Engineering, Henan University, Kaifeng 475004, China; E-Mails: Lijinghua@henu.edu.cn (J.-H.L.); Liqian426@163.com (Q.L.)

**Keywords:** naphthalimide, diamine conjugates, synthesis, antitumor activity, spectroscopy

## Abstract

Eleven novel naphthalimide-diamine conjugates were synthesized and their structures were confirmed by elemental analysis, ^1^H-NMR, ^13^C-NMR and MS. Their *in vitro* antitumor activities were assessed using MTT assays on two cancerous cell lines K562, HCT116, and one normal hepatoma cell line QSG 7701. Compound **7f** exhibited potent antitumor activity on HCT116 cells and favorable cell selectivity toward QSG 7701 compared with the positive control, amonafide. Moreover, **7f** could block HeG2 cells in the G2/M phase and induce HeG2 cells apoptosis. The interaction of compound **7f** with herring sperm DNA was studied by UV/vis absorption and fluorescence spectroscopy under physiological conditions (pH = 7.4). The observed spectral quenching of compound **7f** by DNA and the displacement of EB from DNA-EB complex by compound **7f** indicated that compound **7f** could intercalate into DNA base pairs, which was also corroborated by the effect of KI on compound-DNA interaction. Further caloric fluorescent tests revealed that the quenching mechanism was a static type. Meanwhile, the binding constants, thermodynamic parameters and the effect of NaCl on compound-DNA interaction showed that the type of interaction force was mainly hydrogen bonds and the binding process was driven by hydrogen and van der Waals bonding.

## 1. Introduction

As a common chemical motif, naphthalimides are among a growing class of compounds with desirable anticancer activity. Numerous mono- or bis-naphthalimide derivatives displayed potent antitumor properties against a variety of murine and human tumor cells [1,2,3,4], and some of them such as mitonafide [5], amonafide [6], azonafide [7], DMP-840 [8,9] and Lu-79553 [10] have been tested in clinic trials for the treatment of solid tumors. However, most clinical trials have failed because of a poor therapeutic index, poor water-solubility or dose-limiting bone marrow toxicity such as in the case of mitonafide [11]. Subsequent efforts to improve therapeutic properties of naphthalimides have been made by modifying the naphthalimide skeleton [12,13,14,15,16,17,18,19,20,21,22,23,24,25].

Previous studies revealed that polyamines are a kind of promising carriers to transport cytotoxic agents into cancer cells [26]. Polyamines are important for tumor cell growth and function, the biosynthetic pathway of native polyamines (putrescine, spermidine and spermine) has been a popular target for therapeutic intervention during the last decades [27].

**Figure 1:**
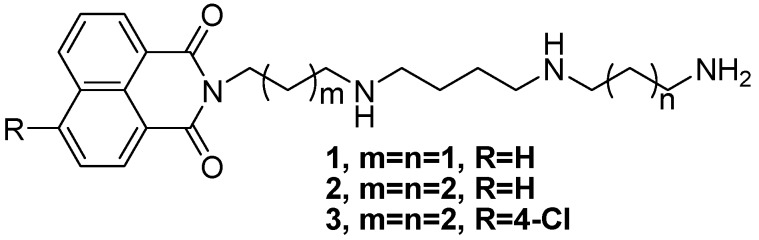
The structures of compounds **1–3**.

Our previous work proved that conjugates **1**–**3** (Figure 1), composed of 1,8-naphthalimide units covalently attached to a polyamine such as spermidine or homospermidine, possessed remarkable cell selectivity through to human hepatoma Bel-7402 and human normal hepatocyte QSG-7701 trials [28,29]. In addition, naphthalimide-polyamine conjugates have been proved to induce cancer cell apoptosis *via* different pathways [28,29,30]. These results encouraged us to screen more substituted naphthalimide-polyamine conjugates in order to assess their antitumor activity*.*

DNA as carrier of genetic information is a major target for drug interaction due to its abilities to interfere with transcription (gene expression and protein synthesis) and DNA replication, a major step in cell growth and division. Generally, a variety of small molecules interact reversibly with DNA in three primary ways, including intercalation of planar or approximately planar aromatic ring systems between base-pairs [31], groove binding in which the small molecules bound on nucleic acids are located in the major or minor groove [18,19] and binding along the exterior of DNA helix through interactions which are generally nonspecific and are primarily electrostatic. The 1,8-naphthalimide derivatives are the DNA intercalating agents because they consist of a flat, generally p-π deficient aromatic system of which binds to DNA by insertion between the base pairs of the double helix. However, there are rare reports on the interaction mechanism of naphthalimide-polyamine conjugates and DNA. In this work, naphthalimide-diamine conjugates were synthesized and their antitumor activity assessed *in vitro.* The interactions between a representative compound **7f** and herring sperm DNA were first studied by UV and ﬂuorescence spectroscopy. The binding constants and main sorts of binding force were also investigated. Moreover, mechanism of how the novel conjugate **7f** killed HeG2 cells **7f** was reported.

## 2. Results and Discussion

### 2.1. Chemistry

**Scheme 1:**
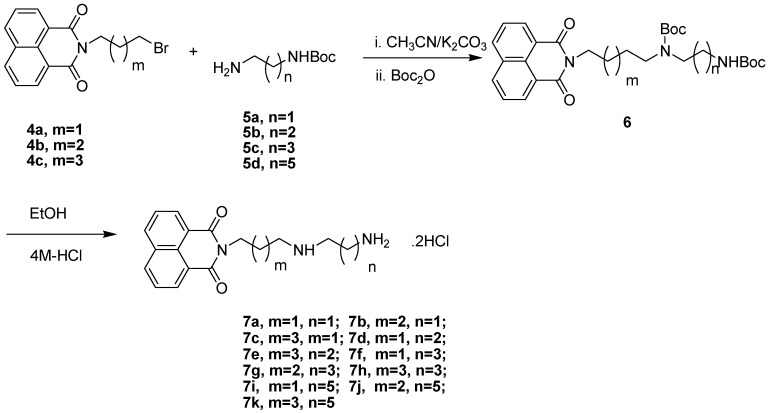
Synthesis of naphthalimide-diamine conjugates.

The synthetic route to the naphthalimide-diamine conjugates **7a**–**k** is shown in Scheme 1. N-(3-bromopropyl)-1,8-naphthalimide (**4a**), N-(4-bromobutyl)-1,8-naphthalimide (**4b**) and N-(5-bromo- pentyl)-1,8-naphthalimide (**4c**) were prepared routinely from 1,8-naphthalimide and 1,4-dibromobutane (or 1,3-dibrompropane, 1,5-dibromopentane) in the presence of potassium carbonate and potassium iodide. The Boc-protected diamines **5** were prepared according to a modified procedure reported previously [32]. The N-alkylation reactions of **5** with **4** in the presence of potassium carbonate in dry acetonitrile at 45 °C generated intermediates which were difficult to purify because of their instability [33]. Without separation, N-Boc protection with Boc_2_O led to the formation of stable intermediates **6**. Subsequently the Boc groups were removed with 4M HCl at room temperature to provide target compounds **7** as dihydrochloride salts in yields of 50%–80%. The structures of target compounds **7** were confirmed by ^1^H-NMR, ^13^C-NMR, ESI-MS, and elemental analysis.

### 2.2. Cytotoxic Effects

**Figure 2:**
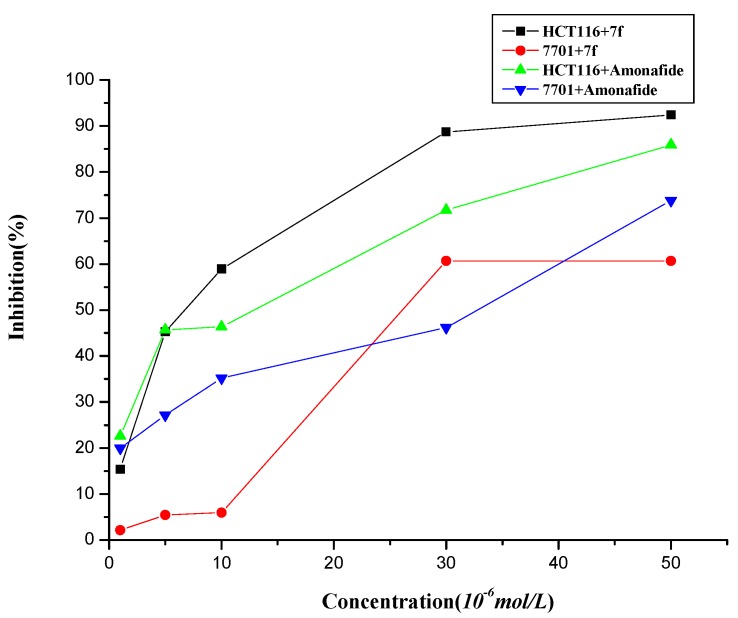
The proliferation inhibition of **7f** and amonafide assayed by MTT.

The cytotoxicities of the novel conjugates were assessed *in vitro* by the MTT assay in the presence of aminoguanidine (an inhibitor of amine oxidase) against three cell lines, human leukemia K562, cancer of colon HCT116 and human normal hepatocyte QSG-7701 [34]. As is shown in Table 1, most of the naphthalimide-diamine conjugates **7a**–**k** showed good antitumor activities against K562, HCT116 cells, in which compound **7f** seemed to have equipotent antitumor activity against HCT116 and cell selectivity toward QSG-7701 compared to the control, amonafide. At the same time, compounds **7f–h**, which have putrescine backbones, exhibited good antitumor activities against K562, HCT116 cells. In addition, compounds **7j–k**, which have longer side chains than the others, also exhibited good antitumor activities. Moreover, The compound **7f** with a primary terminal amino group in the polyamine motif exhibited better biological properties than the corresponding compounds with a terminal tertiary amino group [35]. However, the dose-responsive curves shown in Figure 2 indicated that compound **7f** displayed much better selectivity between HCT116 and QSG-7701 than amonafide, especially at the concentrations around the IC_50_ values (5–10 μM). Thus, compound **7f** was selected for further investigation.

**Table 1:** .*In vitro* activity of compound naphthalimide-diamine conjugates (**7a**–**k**).

Compd.	IC_50_(µM)		
	K562	HCT116	7701
**7a**	48.76 ± 3.93	19.33 ± 4.39	>50
**7b**	25.60 ± 3.74	13.93 ± 2.68	33.89 ± 4.32
**7c**	38.75 ± 1.76	9.89 ±1.11	>50
**7d**	19.71 ± 7.23	8.95 ± 3.87	8.04 ±3.35
**7e**	22.36 ± 1.21	9.52 ± 3.31	22.16 ± 0.67
**7f**	18.87 ± 1.97	5.45 ± 1.71	19.69 ± 3.42
**7g**	14.03 ± 3.32	13.88 ± 2.54	11.97 ± 4.07
**7h**	15.89 ± 1.53	16.21 ± 4.28	18.78 ± 4.94
**7i**	41.35 ± 1.76	10.97 ± 1.71	>50
**7j**	12.56 ± 0.81	16.44 ±1.75	13.13 ± 3.94
**7k**	15.89 ± 1.83	9.95 ± 1.81	14.70 ± 3.91
**Amonafide**	10.10 ± 2.59	6.86 ± 1.89	20.29 ± 2.43

All data are expressed as means ± SD from three separate determinations. IC_50_ values were given only if they were less than 50 µM, which was the maximum concentration tested.

### 2.3. Cell Cycle Analysis and Apoptosis

**Figure 3:**
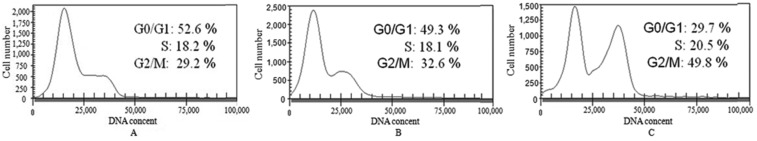
Arrest of cell cycle progress in HepG2 cells treated with **7f** for 48 h. Cells were fixed with ethanol and stained with PI. Cell cycle distribution was analyzed by HCS. **(A)** Control; **(B)** Compound **7f**: 10 μM; **(C)** Compound **7f**: 20 μM.

To reveal the cytotoxic mechanism of compound **7f**, we ﬁrst examined its effects on the cell cycle perturbation. The DNA content analysis by High Content Screening (HCS) confirmed that **7f** could induce HepG2 cell cycle perturbation (Figure 3). The exposure of HepG2 cells to **7f** resulted in the dose-dependent accumulation of cells in G2/M phase from 29.7% of the control to 49.8% of **7f**. Meanwhile, the corresponding reduction of G0/G1 phase from 52.6% of the control to 29.7% of **7f** was also observed, accompanying by the little change in the S phase. The results showed that compound **7f** could block HepG2 cells in the G2/M phase.

**Figure 4:**
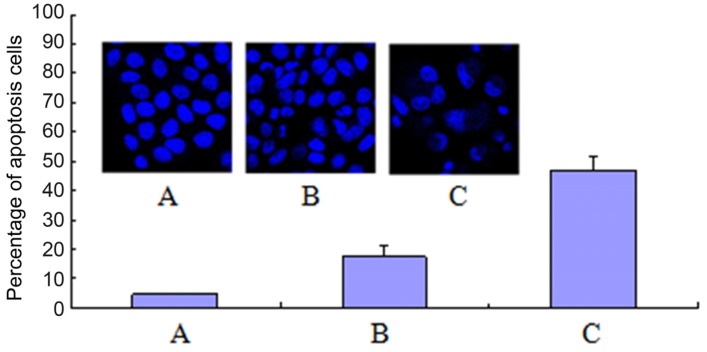
**7f** induced HepG2 cell apoptosis. (**A**) Control; (**B**) Compound **7f**: 10 μM; (**C**) Compound **7f**: 20 μM.

Apoptosis, or namely programmed cell death, can be triggered by several stimuli. Both naphthalimides and polyamine derivatives could trigger cell apoptosis [36]. In order to determine whether the antitumor-proliferative effect of compound **7f** was associated with cell apoptosis, the HepG2 cell apoptosis was detected by the staining of DNA with Hoechst 33342, and the apoptotic cells were counted by selecting 200 cells randomly (Figure 4). The apoptotic bodies were observed clearly in **7f**-treated groups by laser scanning confocal microscope. **7f** could trigger apoptosis in a dose-dependent manner with the 47.0% apoptotic ratio of **7f** at 20 μM compared with the less than five percent in the control group.

### 2.4. UV Spectroscopy

**Figure 5:**
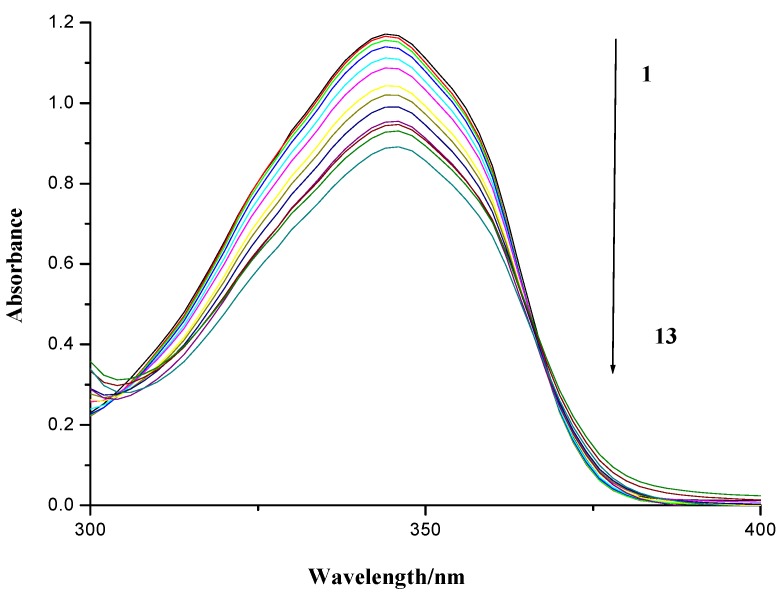
UV absorption spectra of compound **7f** with herring sperm DNA. Numbers 1–13 indicated the DNA concentration: 0.0, 4.56 × 10^−6^, 9.13 × 10^−6^, 13.69 × 10^−6^, 27.4 × 10^−6^, 41.08 × 10^−6^, 54.77 × 10^−6^, 68.46 × 10^−6^, 82.15 × 10^−6^, 95.84 × 10^−6^, 109.54 × 10^−6^, 123.23 × 10^−6^ and 136.92 × 10^−6^ mol∙L^−^^1^, respectively. The concentration of compound **7f** applied were 80 × 10^−6^ mol∙L^−1^.

As is shown in Figure 5, the UV spectrum of compound **7f** in the absence and presence of herring sperm DNA was measured by an ultraviolet visible range spectrophotometer. It was observed that a continuous decrease in the absorbance of compound **7f** followed with the increasing concentration of DNA, implying compound **7f** could insert into the base pairs of DNA. The spectral effects have been rationalized that the empty π*-orbital of the small molecule couples with the π*-orbital of the DNA base pairs, which causes an energy decrease and a decrease of the π–π* transition energy [37,38,39]. At the same time, the empty π*-orbital is partially ﬁlled with electrons to reduce the transition probability. Therefore, the absorption of small molecules should exhibit hypochromism.

**Figure 6:**
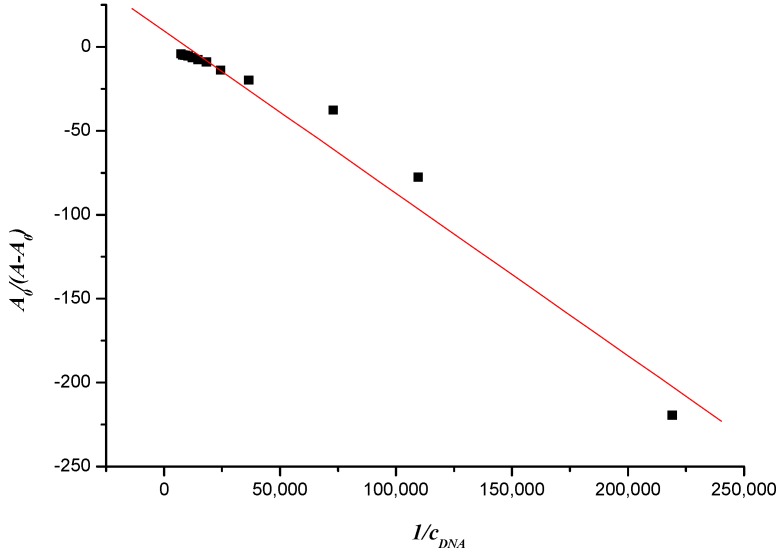
Plot of *A_0_*/(*A* −*A_0_*) *versus1/c_DNA_* of the interaction between compound **7f** and herring sperm DNA (10^−6^ mol∙L^−1^).

Utilizing the absorption spectrum obtained by UV, we could also calculate compound **7f**’s apparent binding constant according to the following formula [40]:

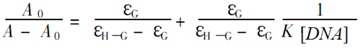
(1)
where *A_0_* and *A* denote the absorbance in the absence and presence of DNA, respectively and where *ε_G_*and *ε_ H–G_* denote the molar absorption coefficient of compound and its formed complex with DNA. The value of apparent binding constant could be measured from the intercept and slope by plotting *A_0_*/(*A* − *A_0_*) against *c*_DNA_, and the corresponding value of *K* was 9.806 × 10^3^ (Figure 6).

### 2.5. Fluorescence Spectroscopy

#### 2.5.1. Fluorescence Quenching

To evaluate the DNA binding properties of naphthalimide homospermidine conjugate, the inherent ﬂuorescence of compound **7f** allowed us to investigate its interaction with herring sperm DNA by fluorescence spectrometry. 

**Figure 7:**
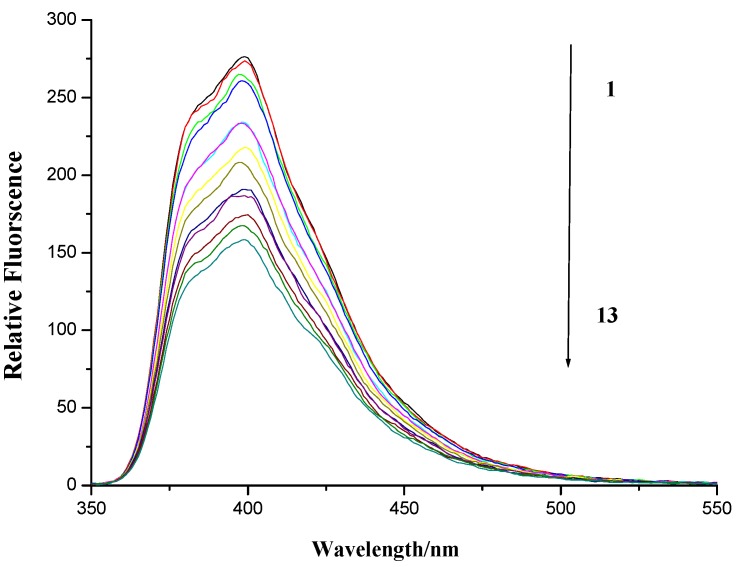
Fluorescence spectroscopy of compound **7f** and herring sperm DNA. Numbers 1–13 indicated the DNA concentration: 0.0, 4.56 × 10^−6^, 9.13 × 10^−6^, 13.69 × 10^−6^, 27.4 × 10^−6^, 41.08 × 10^−6^, 54.77 × 10^−6^, 68.46 × 10^−6^, 82.15 × 10^−6^, 95.84 × 10^−6^, 109.54 × 10^−6^, 123.23 × 10^−6^ and 136.92 × 10^−6^ mol∙L^−1^, respectively. Compound **7f** applied was 80 × 10^−6^ mol∙L^−1^. Scan condition of compound **7f**: EX = 345nm, EM = 355~600 nm; Slits of both EX and EM were 5 nm and 2.5 nm, respectively.

As is shown in Figure 7, the ﬂuorescence of compound **7f** was quenched upon the addition of DNA. This indicated that DNA is one potential target of compound **7f** as expected.

**Figure 8:**
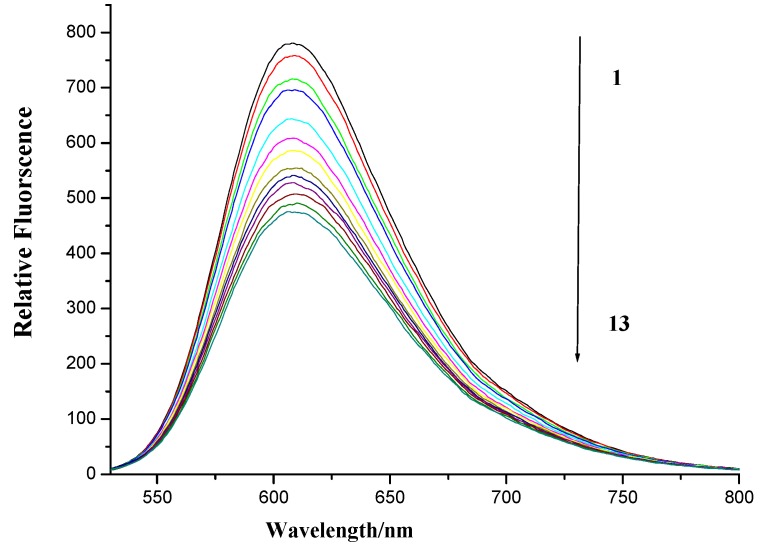
Fluorescence spectroscopy of compound **7f** with herring sperm DNA-EB. Numbers 1–13 indicated the compound **7f** concentration: 0.0, 4 × 10^−6^, 8 × 10^−6^, 12 × 10^−6^, 24 × 10^−6^, 36 × 10^−6^, 48 × 10^−6^, 60 × 10^−6^, 72 × 10^−6^, 84 × 10^−6^, 96 × 10^−6^, 108 × 10^−6^ and 120 × 10^−6^ mol∙L^−1^, respectively. DNA and EB applied was 13.7 × 10^−6^ and 15.7 × 10^−6^ mol∙L^−1^, respectively. Scan condition: EX = 510 nm, EM = 520~800 nm; Slits of both EX and EM were 5 nm and 10 nm, respectively.

Ethidium bromide (EB) is a well known DNA intercalator, which is often used as a spectral probe to establish the mode of binding of small molecules to double-helical DNA [41]. The ﬂuorescence of EB increases after binding with DNA due to intercalation. Like EB, if naphthalimides intercalate into the helix of DNA, it would compete with EB for its intercalation sites in DNA, and the displacement of EB from the DNA–EB complex leads to a signiﬁcant decrease in the ﬂuorescence intensity of the DNA-EB complex [42,43,44]. Therefore, herring sperm DNA-EB complex in the presence of increasing concentrations of naphthalimide-diamine conjugate **7f** was also measured. As is shown in Figure 8, the ﬂuorescence intensity of DNA-EB complex was decreased by gradually growing concentrations of compound **7f**, suggesting that compound **7f** could intercalate into DNA and a new complex was possibly formed between compound **7f** and DNA [45].

#### 2.5.2. Fluorescence Quenching Mechanism

Fluorescence quenching can occur by different mechanisms, which are usually classiﬁed as dynamic quenching and static quenching. Dynamic quenching refers to a process whereby the ﬂuorophore and the quencher come into contact during the transient existence of the excited state, so the bimolecular quenching constants would be larger at higher temperatures. Static quenching, however, results from the formation of a ground state complex between the ﬂuorophore and the quencher, which decreases [46]. To elucidate the quenching mechanism of the interaction between naphthalimide-diamine conjugate and DNA (or DNA-EB), ﬂuorescence quenching tests were also performed at 298, 303 and 310 K, which could be described by Stern-Volmer equation [47,48,49].

**Figure 9:**
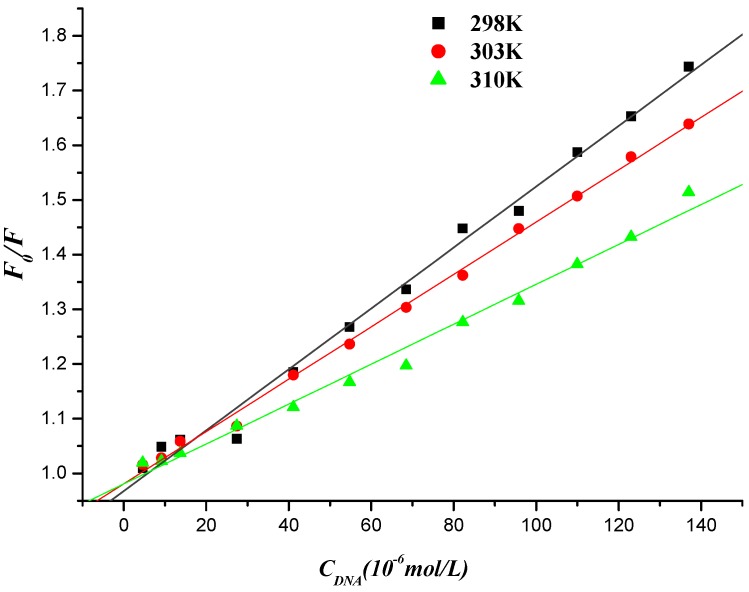
Stern-Volmer plot of fluorescence quenching of compound **7f** by herring sperm DNA at different temperatures.

**Figure 10:**
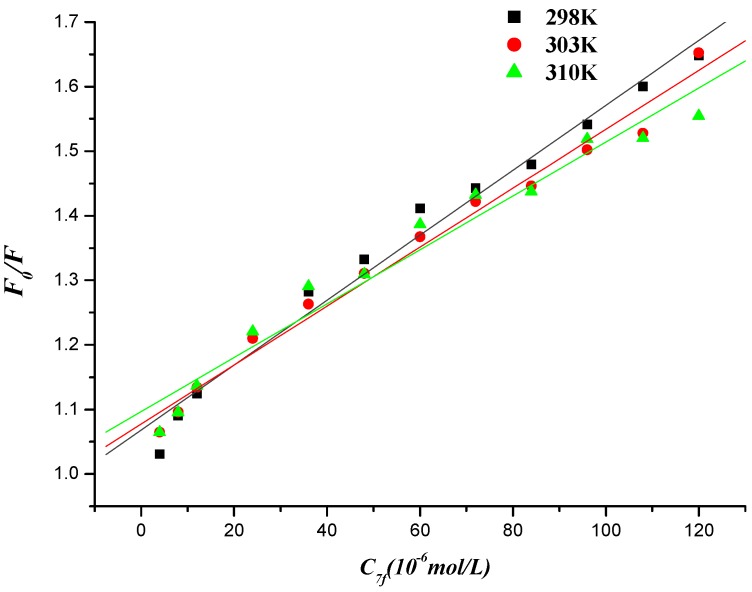
Stern-Volmer plot of fluorescence quenching of herring sperm DNA-EB by compound **7f** at different temperatures.

The Stern–Volmer equation is the following:

*F_0_*/*F* = 1+ *K*_SV_*c* = 1+ *K* τ*_0_c* (2)

where *F_0_* and *F* are the ﬂuorescence intensities in the absence and presence of quencher (DNA for compound **7f** or compound **7f** for DNA-EB, respectively), *K_SV_* is the Stern-Volmer quenching constant, [*c*] is the concentration of DNA (or compound **7f**), *K_q_* is the biomolecule quenching rate constant and *Kq* = *K_SV_*/τ*_0_*. τ*_0_* is the average lifetime of the molecule without any quencher and the ﬂuorescence lifetime of the biopolymer is 10^−8^ s [50]. The Stern–Volmer plots of *F_0_*/*F versus* [*c*] at the three temperatures were shown in Figure 9 and Figure 10, and the calculated *K_SV_* and *K_q_* values were presented in Table 2 and Table 3.

**Table 2:** Quenching constants of the interaction between compound **7f** and herring sperm DNA at different temperatures.

*T*/K	*K*_SV_/(L∙mol^−1^)	*K*_q_/(L∙mol^−1^)	*R*
298	5.567 × 10^3^	5.567 × 10^11^	0.9956
303	4.789 × 10^3^	4.789 × 10^11^	0.9987
310	3.651 × 10^3^	3.651 × 10^11^	0.9945

**Table 3:** Quenching constants of the interaction between compound **7f** and herring sperm DNA-EB at different temperatures.

*T*/K	*K*_SV_/(L∙mol^−1^)	*K*_q_/(L∙mol^−1^)	*R*
298	5.028 × 10^3^	5.028 × 10^11^	0.9907
303	4.564 × 10^3^	4.654 × 10^11^	0.9922
310	4.176 × 10^3^	4.176 × 10^11^	0.9809

The values of the quenching constant *K_SV_* decreased with increasing temperature and the values of *K_q_* were much greater than that of the maximum scattering collision quenching constant (2.000 × 10^10^ L∙mol^−1^), indicating that the ﬂuorescence quenching of compound **7f** initiated by DNA or DNA-EB complex initiated by compound **7f** was static quenching [51].

#### 2.5.3. Interaction Mode between Compounds and DNA

When small molecules bind independently to a set of equivalent sites in a macromolecule, the binding constant (*K_b_*) can be determined by the following equation [40,52]:

log[1/*c*] = log [*F*/(*F*_0_ − *F*)] + log*K_b_* (3)

where *K_b_* denotes the binding constant for interaction of naphthalimide–DNA, and *F_0_*, *F*, [*c*] have the same meanings as in Equation (2).

**Figure 11:**
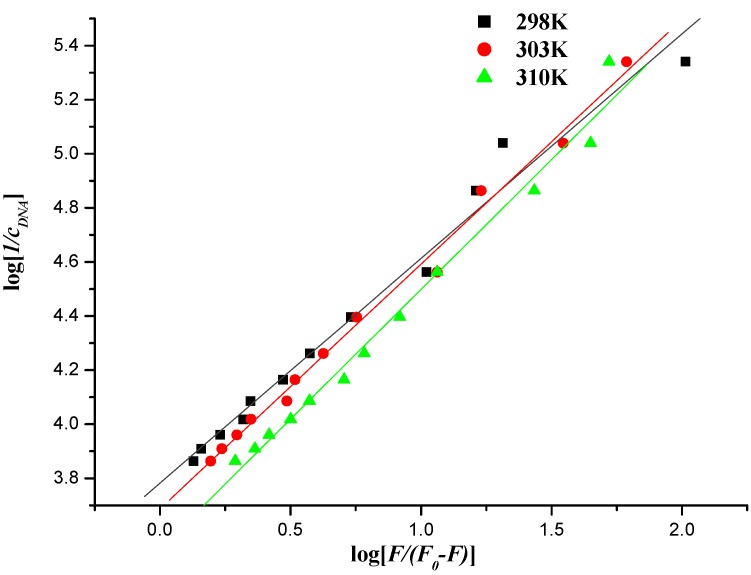
Plot of log [*1*/*c_DNA_*] *versus* log [*F*/(*F*_0_ − *F*)] of the interaction between compound **7f** and herring sperm DNA at different temperatures.

**Figure 12:**
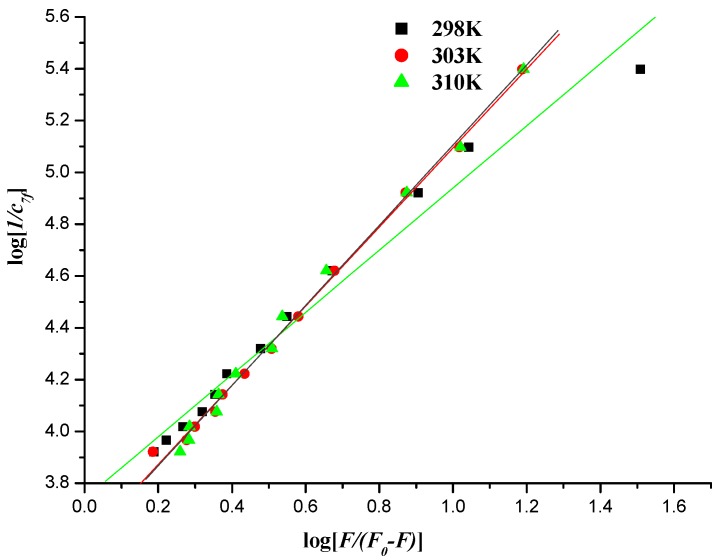
Plot of log [*1*/*c_7f_*] *versus* log [*F*/(*F*_0_ − *F*)] of the interaction between compound **7f** and herring sperm DNA-EB at different temperatures.

**Table 4:** Binding constants and thermodynamic parameters of the interaction between compound **7f** and herring sperm DNA at different temperatures.

*T/K*	*K_b_*/(kJ∙mol^−1^)	∆*G°*/(kJ∙mol^−1^)	∆*H°*/(kJ∙mol^−1^)	∆*S°*/(kJ∙mol^−1^)	*R*
298	6.059 × 10^3^	−21.578	−32.983	−0.0383	0.9897
303	4.864 × 10^3^	−21.387	−32.983	−0.0383	0.9965
310	3.462 × 10^3^	−21.004	−32.983	−0.0383	0.9923

**Table 5:** Binding constants and thermodynamic parameters of the interaction between compound **7f** and herring sperm DNA-EB at different temperatures.

*T/K*	*K_b_*/(kJ∙mol^−1^)	∆*G°*/(kJ∙mol^−1^)	∆*H°*/(kJ∙mol^−1^)	∆*S°*/(kJ∙mol^−1^)	*R*
298	5.482 × 10^3^	−21.330	−59.229	−0.127	0.9884
303	3.695 × 10^3^	−21.172	−59.229	−0.127	0.9982
310	3.611 × 10^3^	−21.112	−59.229	−0.127	0.9975

The values of *K_b_* could be measured from the intercept and slope by plotting log [1/*c*] against log [*F*/(*F_0_* − *F*)] (intercept = log *K_b_*) (Figure 11 and Figure 12), and the corresponding values of *K_b_* were listed in Table 4 and Table 5. The down-regulated trend of *K_b_* with increasing temperature was in accordance with *K_SV_’*s dependence on temperature as mentioned above, implying that the binding between naphthalimide and DNA was moderate, and a reversible naphthalimide-DNA complex might be formed [53].

There are several acting forces between a small organic molecule and biomacromolecules, such as hydrophobic force, hydrogen bond, van der Waals force, electrostatic interactions, *etc*. It is assumed that the interaction enthalpy change (∆*H*°) does not vary signiﬁcantly over the limited temperature range studied, thus the thermodynamic parameters can be calculated from the van’t Hoff equation:

ln (*K*_2_/*K*_1_) = (1/*T*_1_ − 1/*T*_2_) ∆*H*°/*R* (4)

∆*G*° = −*RT* ln*K =* ∆*H*° − *T*∆*S*° (5)

In Equations (4) and (5), *K* is analogous to the binding constant at the corresponding temperature and *R* is gas constant. The enthalpy change (∆*H*°) and entropy change (∆*S*°) were calculated from the Equations (4) and (5), and the corresponding results were listed in Table 4 and Table 5. From Table 4 and Table 5, it can be seen that the negative ∆*H*° and negative ∆*S*° values showed that the hydrogen bond and weak van der Waals played a dominant role in the interactions between compound **7f** and DNA [54].

Kenaka [55] found that hydrogen bonding was the main feature of DNA intercalating agents, and the present evidences indicated that compound **7f** was a DNA intercalator. Therefore, it was inferred that the process of interaction between compound **7f** and DNA was driven by hydrogen bonding and van der Waals forces. It was also inferred that planar structure of naphthalene ring intercalated into the DNA base pairs when compound **7f** bound to DNA between the double helix and that the hydrogen bond and weak van der Waals played a dominant role in the interactions between compound **7f** and DNA.

#### 2.5.4. Iodide Quenching Studies

A highly negatively charged quencher is expected to be repelled by the negatively charged phosphate backbone of DNA, therefore an intercalative bound drug molecules should be protected from being quenched by anionic quencher, but the free aqueous complexes or groove binding drugs should be quenched readily by anionic quenchers. At the same time, whether the quencher accesses to fluorophore also plays a role in free and bound one [56].

**Figure 13:**
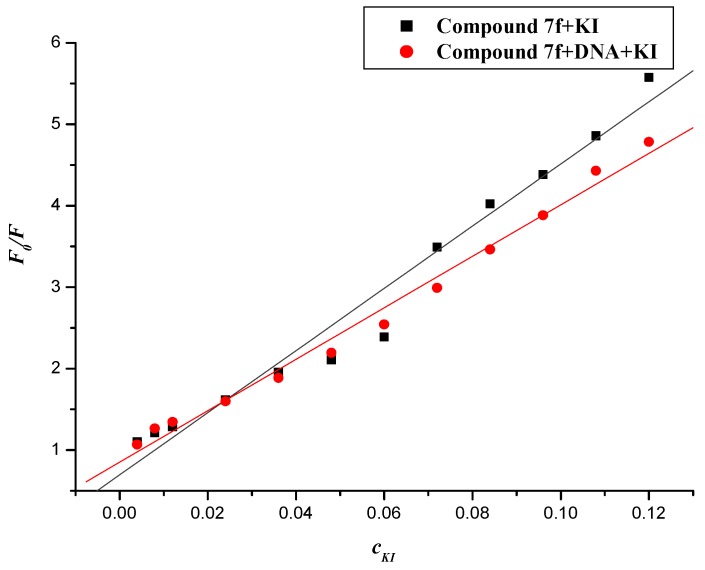
Fluorescence quenching plots of compound **7f**with KI in the absence and presence of DNA. *c*(**7f**) = 20 × 10^−6^ mol∙L^−1^; *c*(DNA) = 22.84 × 10^−6^ mol∙L^−1^; the KI concentration was 4~120 × 10^−3^ mol∙L.

Negatively charged I^−^ was selected for this purpose. The quenching constants (*K_sv_*) were obtained from the Stern–Volmer equation. The values of *K_sv_* of compound **7f** with I^−^ ion in the absence and presence of DNA were 38.178 and 31.575 (L∙mol^−1^), respectively (shown in Figure 13). It was apparent that iodide quenching effect was decreased when compound **7f** was bound to DNA, which suggested that the compound **7f** is likely intercalated into the base pairs of DNA.

#### 2.5.5. Effect of Ionic Intensity on the Compound 7f and DNA Interaction

**Figure 14:**
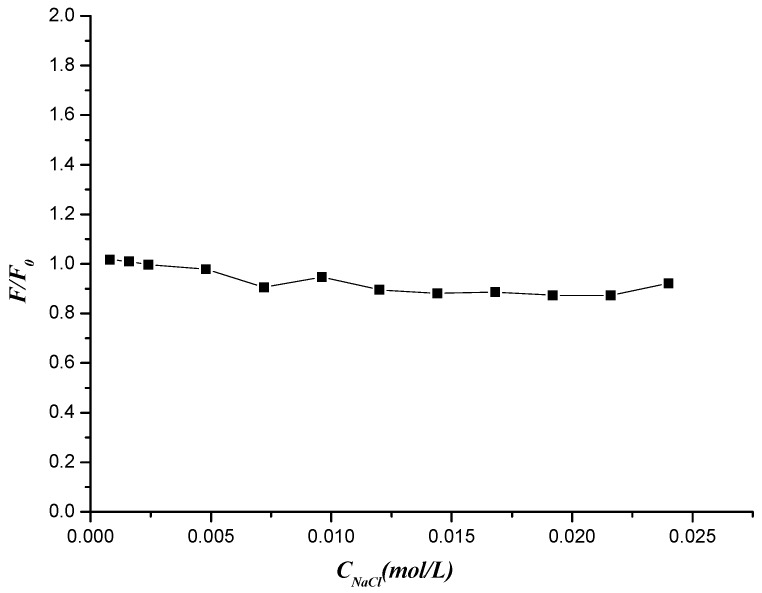
Effects of NaCl on the fluorescence intensity of compound **7f**-DNA system.

DNA is an anionic polyelectrolyte with phosphate groups and monitoring the spectral change with different ionic strength is an efficient method to distinguish the binding modes between molecules and DNA. NaCl is used to control the ionic strength of the solutions. The addition of Na^+^ would attenuate the electrostatic interaction between molecules and DNA because of its competition for phosphate groups in DNA [57]. Hence the effect of NaCl on the ﬂuorescence of DNA–compound **7f** system was studied. As is seen from Figure 14, the fluorescence intensity of compound **7f-**DNA complex was basically unchanged with increasing concentration of NaCl. The results revealed that interaction between compound **7f** and DNA could exclude the electrostatic interaction mode and was prompted by hydrogen bond and van der Waals force.

## 3. Experimental Section

### 3.1. General Information

All chemicals (reagent grade) used were commercially available. All the ^1^H-NMR and ^13^C-NMR spectra were recorded on a Bruker AV-400 model spectrometer in D_2_O. Chemical shifts (*δ*) for ^1^H-NMR spectra were reported in parts per million to residual solvent protons. ESI-MS spectra were recorded on an ESQUIRE-LC Mass spectrometer. Elemental analyses were performed on a Gmbe VarioEL Elemental instrument and were within 0.4% of the theoretical values.

### 3.2. Synthesis of Naphthalimide-Diamine Conjugates

The Boc-protected amine **5** (3 mmol) was dissolved in anhydrous acetonitrile (30 mL), and solid K_2_CO_3_ (0.69 g, 5 mmol) was added. After the mixture was stirred for 15 minutes at ambient temperature, *N*-(3–bromopropyl)-1,8-naphthalimide (**4a**) (or *N*-(4–bromobutyl)-1,8-naphthalimide (**4b**) and *N*-(5–bromopentyl)-1,8-naphthalimide **(4c**), 2 mmol) in acetonitrile (10 mL) was added dropwise with constant stirring at 45 °C, then the reaction mixture was stirred overnight. The volatiles were removed under vacuum to give a residue which was redissolved in CHCl_3_ (30 mL) and washed with aqueous Na_2_CO_3_ (10%, *w*/*v*). The organic phase was separated, dried over anhydrous Na_2_SO_4_, filtered, and concentrated. The above residue was dissolved in methanol (30 mL), a solution of di-*tert*-butyl dicarbonate (3 mmol) in methanol (10 mL) was added dropwise and stirred at ambient temperature overnight. The solvents were removed under reduced pressure to give a residue, which was redissolved in CHCl_3_ (30 mL) and washed with water. The organic phase was separated, dried over anhydrous Na_2_SO_4_, filtered, and concentrated. The residue was subjected to flash chromatography (20% Petroleum /EtOAc, *v*/*v*, R_f_ = 0.25) to obtain the Boc protected intermediates **6**.

The respective N-Boc-protected amine (1.2 mmol) was dissolved in EtOH (20 mL) and stirred at 0 °C for 10 min. 4 M HCl (15 mL) was added dropwise at 0 °C. The reaction mixture was stirred at room-temperature overnight. The solution typically gave a bright white solid as a precipitate. The solid was filtered, washed several times with absolute ethanol and dried under vacuum to give the pure target compounds **7**.

*2-[3-(2-Aminoethylamino)propyl]1H-benz-[de]isoquinoline-1,3(2H)-dione dihydrochloride* (**7a**). Yield: 76.3%; m.p.: 233.4–235.1 °C; ^1^H-NMR (D_2_O) *δ*: 7.37 (d, 2H, *J* = 8.0 Hz), 7.33 (d, 2H, *J* = 7.2 Hz), 7.0 (t, 2H, *J* = 7.6 Hz), 3.55 (t, 2H,*J* = 7.2 Hz), 3.46 (s, 4H), 3.15 (t, 2H,*J* = 8.0 Hz), 1.83 (t, 2H,*J* = 6.8 Hz); ^13^C-NMR (D_2_O) *δ*: 163.75, 134.73, 130.76, 129.54, 126.52, 125.10, 118.75, 45.79, 44.35, 37.16, 35.50, 24.11; ESI-MS *m*/*z*: 298.2 (M+H-2HCl)^+^; Anal. calcd. for C_17_H_21_Cl_2_N_3_O_2_ C, 55.14; H, 5.72; N, 11.35; found: C, 54.83; H, 5.48; N, 10.94.

*2-[4-(2-Aminoethylamino)butyl]1H-benz-[de]isoquinoline-1,3(2H)-dione dihydrochloride* (**7b**). Yield: 58.1%; m.p.: 243.1–244.1 °C; ^1^H-NMR (D_2_O) *δ*: 7.39 (d, 2H, *J* = 8.0 Hz), 7.00 (d, 2H, *J* = 7.2 Hz), 7.12 (t, 2H, *J* = 7.6 Hz), 3.43–3.47 (m, 6H), 3.18 (t, 2H, *J* = 8.0 Hz), 1.71–1.74 (m, 2H), 1.45–1.46 (m, 2H); ^13^C-NMR (D_2_O) *δ*: 163.84, 134.60, 130.69, 129.59, 126.48, 125.17, 118.95, 47.60, 44.13, 39.47, 35.49, 23.99, 23.18; ESI-MS *m*/*z*: 312.2 (M+H-2HCl)^+^; Anal. calcd. for C_18_H_23_Cl_2_N_3_O_2_·0.5H_2_O: C, 56.26; H, 6.03; N, 10.93; found: C, 56.18; H, 5.76; N, 10.66.

*2-[5-(2-Aminoethylamino)pentyl]1H-benz-[de]isoquinoline-1,3(2H)-dione dihydrochloride* (**7c**). Yield: 63.7%; m.p.: 233.8–235.1 °C; ^1^H-NMR (D_2_O) *δ*: 7.42 (d, 2H, *J* = 8.0 Hz), 7.35 (d, 2H, *J* = 7.2 Hz), 7.02 (t, 2H, *J* = 7.6 Hz), 3.40–3.45 (m, 6H), 3.15 (t, 2H, *J* = 8.0 Hz), 1.73–1.77 (m, 2H), 1.36–1.38 (m, 4H); ^13^C-NMR (D_2_O) *δ*: 163.89, 134.54, 130.67, 129.64, 126.46, 125.23, 119.06, 48.08, 44.12, 39.86, 35.48, 26.40, 25.10, 23.21; ESI-MS *m*/*z*: 326.2 (M+H-2HCl)^+^; Anal. calcd. for C_19_H_25_Cl_2_N_3_O_2_·0.3H_2_O: C, 56.52; H, 6.39; N, 10.41; N, 10.09; found: C, 56.70; H, 6.10; N, 10.13.

*2-[3-(3-Aminopropylamino)propyl]1H-benz-[de]isoquinoline-1,3(2H)-dione dihydrochloride* (**7d**). Yield: 68.8%; m.p.: 233.1–234.2 °C; ^1^H-NMR (D_2_O) *δ*: 7.60 (d, 2H, *J* = 8.0 Hz), 7.57 (d, 2H, *J* = 7.2 Hz), 7.18 (t, 2H, *J* = 7.6 Hz), 3.69 (t, 2H,*J* = 7.2 Hz), 3.11–3.24 (m, 6H), 2.14–2.16 (m, 2H), 1.89–1.93 (m, 2H); ^13^C-NMR (D_2_O) *δ*: 164.20, 134.93, 130.98, 129.91, 126.68, 125.55, 119.18, 45.43, 44.72, 37.27, 36.61, 24.16, 23.79; ESI-MS *m*/*z*: 312.2 (M+H-2HCl)^+^; Anal. calcd. For C_18_H_23_Cl_2_N_3_O_2_·0.5H_2_O: C, 56.26; H, 6.03; N, 10.93; found: C, 56.29; H, 5.88; N, 10.69.

*2-[5-(3-Aminopropylamino)pentyl]1H-benz-[de]isoquinoline-1,3(2H)-dione dihydrochloride* **(7e**). Yield: 72.5%; m.p.: 238.6–239.8 °C; ^1^H-NMR (D_2_O) *δ*: 7.36 (d, 2H, *J* = 8.0 Hz), 7.29 (d, 2H, *J* = 7.2 Hz), 6.97 (t, 2H, *J* = 7.6 Hz), 3.36 (t, 2H, *J* = 7.2 Hz), 3.06–3.20 (m, 6H), 2.11–2.15 (m, 2H), 1.70–1.72 (m, 2H), 1.30–1.34 (m, 4H); ^13^C-NMR (D_2_O) *δ*: 163.78, 134.49, 130.60, 129.55, 126.41, 125.12, 118.95, 47.69, 44.51, 39.85, 36.61, 26.40, 25.07, 23.79, 23.26; ESI-MS *m*/*z*: 340.3 (M+H-2HCl)^+^; Anal. calcd. for C_20_H_27_Cl_2_N_3_O_2_·0.3H_2_O: C, 57.50; H, 6.66; N, 10.06; found: C, 57.52; H, 6.30; N, 9.78.

*2-[3-(4-Aminobutylamino)propyl]1H-benz-[de]isoquinoline-1,3(2H)-dione dihydrochloride* (**7f**). Yield: 69.7%; m.p.: 235.1–235.9 °C; ^1^H-NMR (D_2_O) *δ*: 7.59 (d, 2H, *J* = 8.0 Hz), 7.56 (d, 2H, *J* = 7.2 Hz), 7.17 (t, 2H, *J* = 7.6 Hz), 3.66 (t, 2H, *J* = 7.2 Hz), 3.01–3.11 (m, 6H), 1.76–1.88 (m, 6H); ^13^C-NMR (D_2_O) *δ*: 164.00, 134.70, 130.75, 129.69, 126.44, 125.35, 119.97, 46.83, 44.99, 39.58, 37.03, 23.91, 23.72, 22.56; ESI-MS *m*/*z*: 326.2 (M+H-2HCl)^+^; Anal. calcd. for C_19_H_25_Cl_2_N_3_O_2_·0.5H_2_O: C, 56.02; H, 6.43; N, 10.32; found: C, 56.29; H, 6.26; N, 10.69.

*2-[4-(4-Aminobutylamino)butyl]1H-benz-[de]isoquinoline-1,3(2H)-dione dihydrochloride* (**7g**). Yield: 78.6%; m.p.: 226.7–228.3 °C; ^1^H-NMR (D_2_O) *δ*: 7.75 (d, 2H, *J* = 8.0 Hz), 7.72 (d, 2H, *J* = 7.2 Hz), 7.31 (t, 2H, *J* = 7.6 Hz), 3.70 (t, 2H, *J* = 7.2 Hz), 3.11–3.20 (m, 6H), 1.78–1.86 (m, 6H), 1.60–1.63 (m, 2H); ^13^C-NMR (D_2_O) *δ*: 163.92, 134.66, 130.74, 129.68, 126.52, 125.27, 119.04, 47.08, 46.92, 39.52, 38.86, 24.10, 23.97, 23.19, 22.83; ESI-MS *m*/*z*: 340.3 (M+H-2HCl)^+^; Anal. calcd. for C_20_H_27_Cl_2_N_3_O_2_: C, 58.25; H, 6.60; N, 10.19; found: C, 56.14, H, 5.88, N, 10.69.

*2-[5-(4-Aminobutylamino)pentyl]1H-benz-[de]isoquinoline-1,3(2H)-dione dihydrochloride* **(7h**). Yield: 59.6%; m.p.: 236.6–237.9 °C; ^1^H-NMR (D_2_O) *δ*: 7.29 (d, 2H, *J* = 8.0 Hz), 7.21 (d, 2H, *J* = 7.2 Hz), 6.90 (t, 2H, *J* = 7.6 Hz), 3.30 (t, 2H, *J* = 7.2 Hz), 3.03–3.13 (m, 6H), 1.79–1.81 (m, 4H), 1.68–1.71 (m, 2H), 1.27–1.32 (m, 4H); ^13^C-NMR (D_2_O) *δ*: 163.66, 134.44, 130.54, 129.48, 126.38, 125.02, 118.87, 47.54, 46.92, 39.84, 38.88, 26.41, 25.08, 24.00, 23.31, 22.83; ESI-MS *m*/*z*: 354.3 (M+H-2HCl)^+^; Anal. calcd. for C_21_H_29_Cl_2_N_3_O_2_·0.7H_2_O: C, 57.46; H, 6.98; N, 9.57; found: C, 57.39; H, 6.92; N, 9.37.

*2-[3-(6-Aminohexylamino)propyl]1H-benz-[de]isoquinoline-1,3(2H)-dione dihydrochloride* (**7i**). Yield: 60.7%; m.p.: 230.6–231.8 °C; ^1^H-NMR (D_2_O) *δ*: 7.62 (d, 2H, *J* = 8.0 Hz), 7.58 (d, 2H, *J* = 7.2 Hz), 7.19 (t, 2H, *J* = 7.6 Hz), 3.68 (t, 2H, *J* = 7.2 Hz), 3.00–3.10 (m, 6H), 1.88–1.91 (m, 2H), 1.69–1.76 (m, 4H), 1.43-1.47 (m, 4H); ^13^C-NMR (D_2_O) *δ*: 164.22, 134.94, 130.98, 129.94, 126.70, 125.50, 119.22, 47.65, 45.08, 39.38, 37.31, 26.50, 25.36, 25.27, 25.16, 24.16; ESI-MS *m*/*z*: 354.3 (M+H-2HCl)^+^; Anal. calcd. for C_21_H_29_Cl_2_N_3_O_2_·1.0H_2_O: 56.76; H, 7.03; N, 9.46; found: C, 56.85; H, 6.71; N,9.85.

*2-[4-(6-Aminohexylamino)butyl]1H-benz-[de]isoquinoline-1,3(2H)-dione dihydrochloride* (**7j**). Yield: 64.5%; m.p.: 235.9–237.2 °C; ^1^H-NMR (D_2_O) *δ*: 7.57(d, 2H, *J* = 8.0 Hz), 7.52 (d, 2H, *J* = 7.2 Hz), 7.15 (t, 2H, *J* = 7.6 Hz), 3.54 (t, 2H, *J* = 7.2 Hz), 2.98–3.09 (m, 6H), 1.66–1.71 (m, 6H), 1.41–1.48 (m, 6H); ^13^C-NMR (D_2_O) *δ*: 164.11, 134.76, 130.86, 129.87, 126.60, 125.51, 119.27, 47.48, 46.96, 39.53, 39.33, 26.48, 25.37, 25.25, 25.14, 24.15, 23.16; ESI-MS *m*/*z*: 368.3 (M+H-2HCl)^+^; Anal. calcd. for C_22_H_31_Cl_2_N_3_O_2_·1.0H_2_O: C, 57.64; H, 7.26; N, 9.17; found: C, 57.36; H, 7.13; N, 8.85.

*2-[5-(6-Aminohexylamino)pentyl]1H-benz-[de]isoquinoline-1,3(2H)-dione dihydrochloride* (**7k**). Yield: 77.2%; m.p.: 229.1–230.3 °C; ^1^H-NMR (D_2_O) *δ*: 7.34 (d, 2H, *J* = 8.0 Hz), 7.30 (d, 2H, *J* = 7.2 Hz), 6.96 (t, 2H, *J* = 7.6 Hz), 3.33 (t, 2H, *J* = 7.2 Hz), 2.91–2.99 (m, 6H), 1.61–1.63 (m, 6H), 1.27–1.37c (m, 8H); ^13^C-NMR (D_2_O) *δ*: 163.96, 134.59, 130.71, 129.75, 126.52, 125.35, 119.19, 47.51, 47.39, 39.92, 39.40, 26.52, 26.49, 25.38, 25.31, 25.17, 25.11, 23.34; ESI-MS *m*/*z*: 382.3 (M+H-2HCl)^+^; Anal. calcd. for C_23_H_33_Cl_2_N_3_O_2_·0.9H_2_O: C, 58.69; H, 7.45; N, 8.93; found: C, 58.36; H, 7.06; N, 8.60.

### 3.3. Biological Materials and Methods

All chemicals used in bioassay were purchased from Sigma (Beijing, China), unless otherwise indicated. RPMI1640 and fetal calf serum (FCS) were purchased from Gibco (Shanghai, China). Stock solution (10 mM) was prepared in DMSO and diluted to various concentrations with serum-free culture medium.

#### 3.3.1. Cell Culture

Cell lines, K562, 7721 and HCT116 were obtained from American Type Culture Collection (ATCC, Shanghai, China). Cells were cultured in RPMI1640, supplemented with 10% heat-inactivated fetal calf serum (FCS), antibiotics (penicillin, 100 units/mL; streptomycin sulfate, 100 μg/mL) at 37 °C, in an atmosphere of 95% air and 5% CO_2_ under humidified conditions. Aminoguanidine (1 mM) was added as an inhibitor of amine oxidase derived from FCS and had no effect on the various parameters of the cell measured in this study.

#### 3.3.2. MTT Assay

Chemosensitivity was assessed using the 3-(4,5-dimethylthiazol-2-yl)-2,5-diphenyltetrazolium bromide (MTT) assay. Briefly, exponentially growing K562 cells were seeded into 96-well plates at 4000 cells/well and treated with indicated concentrations of samples for 48 h, and then 10 μL of MTT (10 mg/mL) was added. After incubation for 4 h at 37 °C, the purple formazan crystals (*i.e.*, a reduced form of MTT) generated from viable cells were dissolved by adding 100 μL 10% sodium dodecyl sulphate (SDS) in each well. The absorbance of each well was then read at 570 nm.

In addition, exponentially growing HCT116 or 7721 cells were seeded into 96-well plates at 5000 cells/well and allowed to attach overnight, and then 100 μL of MTT (1 mg/mL) was added. After incubation for 4 h at 37 °C, the MTT solution was removed and the remaining formazan crystals were dissolved with 150 μL DMSO in each well. The absorbance of each well was then read at 570 nm.

### 3.4. Cell Cycle Analysis and Apoptosis

#### 3.4.1. Cell Cycle Analysis

Exponentially growing HepG2 cells were seeded in 96 well plates (4 × 10^5^ cells/well), cultured for 24 h and then treated with different concentrations of compound **7f** for 48 h. After incubation for 48 h, cells were washed twice with ice-cold 10% PBS, fixed and permeabilized with ice-cold 70% ethanol at −20 °C overnight. The cells were treated with 50 μg/mL RNase A at room temperature for 30 min after washed with ice-cold PBS, and finally stained with 50 μg/mL propidium iodide (PI) in the dark at 4 °C for 30 min. The distribution of cell cycle phases with different DNA contents was read in image analysis system of high content screening living cells.

#### 3.4.2. Apoptosis

Exponentially growing HepG2 cells were seeded in 96 well plates (6 × 10^4^ cells/well) and treated with indicated concentrations of **7f**. After incubated for 48 h, cells were washed three times with PBS, and finally stained with with 10 μM Hoechst 33342 in the dark at 37 °C for 20 min. They were detected by laser scanning confocal microscope and the apoptotic cells were count by selecting 200 cells randomly.

### 3.5. Spectroscopy Measurement

#### 3.5.1. Apparatus

UV–vis absorption spectra were measured on a Unicam UV 500 spectrophotometer (Beijing, China) using a 1.0 cm cell. Fluorescence measurements were performed with a Cary Eclipse spectroﬂuorimeter (Shanghai, China).

#### 3.5.2. UV–Vis Measurements

2 mL solution of compound **7f** (2.00 × 10^−4^ mol∙L^−1^ in Tris-HCl (pH = 7.4) was mixed with 0.0, 0.10, 0.20, 0.30, 0.60, 0.90 1.20, 1.50, 1.80, 2.10, 2.40, 2.70 and 3.0 mL of herring sperm DNA (2.284 × 10^−4^ mol∙L^−1^) respectively. The mixture was diluted to 5 mL with Tris-HCl (pH = 7.4). Thus, samples were prepared in the concentration of DNA at 0.0, 4.56, 9.13, 13.69, 27.4, 41.08, 54.77, 68.46, 82.15, 95.84, 109.54, 123.23 and 136.92 × 10^−6^ mol∙L^−1^. One contained only compound **7f** (80 × 10^−6^ mol∙L^−1^) as control, the others contained different concentration of DNA but had the same concentration of compound **7f**. All the above solution was shaken for 30 min. at room temperature. 

#### 3.5.3. Fluorescence Measurement

#### 3.5.3.1. Interaction of Compound **7f** with DNA

Preparation of sample is the same as that of UV-Vis measurement. Fluorescence wavelengths and intensity areas of samples were measured at following conditions: EX = 345 nm, EM = 355~600 nm temperature: 298, 303 and 310 K.

#### 3.5.3.2. Interaction of Compound **7f** with DNA-EB Complex

Solution of herring sperm DNA (0.3 mL, 2.284 × 10^−5^ mol∙L^−1^ in Tris-HCl (pH = 7.4) and 0.5 mL EB (1.57 × 10^−5^ mol∙L^−1^) was mixed with 0.0, 0.10, 0.20, 0.30, 0.60, 0.90 1.20, 1.50, 1.80, 2.10, 2.40, 2.70 and 3.00 mL of compound **7f** (2.0 × 10^−4^ mol∙L^−1^) respectively. The mixture was diluted to 5 mL with Tris-HCl (pH = 7.4). Thus, samples were prepared in the concentration of compound **7f** at 0.0, 4.0, 8.0, 12.0, 24.0, 36.0, 48.0, 60.0, 72.0, 84.0, 96.0 and 108.0 and 120.0 × 10^−6^ mol∙L^−1^. One contained only DNA (13.7 × 10^−6^ mol∙L^−1^) and EB (15.7 × 10^−6^ mol∙L^−1^) as control, the others contained different concentration of compound **7f** but had the same concentration of DNA and EB. All the above solution was shaken for 30 min. at room temperature. Fluorescence wavelengths and intensity areas of samples were measured at following conditions: EX = 510 nm, EM = 520~800 nm; temperature: 298, 303 and 310 K.

#### 3.5.3.3. Iodide Quenching

Solution of compound **7f** (0.5 mL, 2.00 × 10^−4^ mol/L and herring sperm DNA 0.5 mL (22.84 × 10^−4^ mol/L) in Tris-HCl (pH = 7.4) were mixed with 0.0, 0.10, 0.20, 0.30, 0.60, 0.90 1.20, 1.50, 1.80, 2.10, 2.40, 2.70 and 3.00 of KI (2.0 × 10^−2^ mol∙L^−1^) respectively. Meanwhile, 0.5 mL solution of compound **7f** (2.00 × 10^−4^ mol/L was only mixed with 0.0, 0.10, 0.20, 0.30, 0.60, 0.90 1.20, 1.50, 1.80, 2.10, 2.40, 2.70 and 3.00 mL of KI (2.0 × 10^−2^ mol∙L^−1^) respectively. The mixture was diluted to 5 mL with Tris-HCl (pH = 7.4). Thus, samples were prepared in the concentration of KI at 0.0, 400, 800, 1,200, 2,400, 3,600, 4,800, 6,000, 7,200, 8,400, 9,600, 10,800, 12,000 × 10^−6^ mol∙L^−1^. One kind of samples contained compound **7f** (20 × 10^−6^ mol∙L^−1^), DNA (22.82 × 10^−6^ mol∙L^−1^) and different concentrations of KI. The other groups of samples contained only different concentration of KI and the same concentration of compound **7f** as the control. All the above solution was shaken for 30 min. at room temperature. Fluorescence wavelengths and intensity areas of samples were measured at following conditions: EX = 345 nm, EM = 355~600 nm.

#### 3.5.3.4. Effect of Ionic Intensity on The Interaction between Compound **7f** and DNA

Solution of compound **7f** (1.0 mL, 2.00 × 10^−4^ mol∙L^−1^) and herring sperm DNA 1.0 mL (2.284 × 10^−4^ mol∙L^−1^) in Tris-HCl (pH = 7.4) were mixed with 0.0, 0.10, 0.20, 0.30, 0.60, 0.90 1.20, 1.50, 1.80, 2.10, 2.40, 2.70 and 3.00 mL of NaCl (4.0 × 10^−2^ mol∙L^−1^) respectively. The mixture was diluted to 5 mL with Tris-HCl (pH = 7.4). Thus, samples were prepared with the concentration of NaCl at 0.0, 800, 1,600, 2,400, 4,800, 7,200, 9,600, 12,000, 14,400, 16,800, 19,200, 21,600 and 24,000 × 10^−6^ mol∙L^−1^. The above mixtures were divided into two groups, in which one contained only compound **7f** (40 × 10^−6^ mol∙L^−1^) and DNA (45.68 × 10^−6^ mol∙L^−1^) as control, the others contained different concentration of NaCl with the same concentration of compound **7f** and DNA. All the above solution was shaken for 30 min. at room temperature. Fluorescence wavelengths and intensity areas of samples were measured at following conditions: EX = 345 nm, EM = 355~600 nm.

## 4. Conclusions

A series of naphthalimide-diamine conjugates were synthesized and their *in vitro* antitumor activities were evaluated. Compound **7f** was found to have potent antitumor activity and good cell selectivity with amonafide as a control. Moreover, compound **7f** could arrest HepG2 cells in the G2/M phase and induce HepG2 cells apoptosis. The interaction of compound **7f** with DNA was first studied by spectroscopic methods. The binding of compound **7f** to DNA resulted in a series of changes in the spectral characteristics. The absorption spectra of compound **7f** with added DNA showed a hypochromic effect and the fluorescence emission of DNA-EB was efficiently quenched by compound **7f**. These observed spectral data and the iodide quenching effect suggested that compound **7f** interacts with DNA through an intercalative mode. Further fluorescent assays at different temperatures disclosed that the quenching mechanism of both compound **7f** with DNA and compound **7f** with DNA-EB was a static type. Meanwhile, the binding constant, thermodynamic parameters obtained from the same caloric fluorescent tests and the effect of NaCl on compound **7f**-DNA interaction suggested that the binding process was driven by hydrogen bonding and van der Waals forces.
